# Supraclavicular Recurrence in Completely Resected (y)pN2 Non-Small Cell Lung Cancer: Implications for Postoperative Radiotherapy

**DOI:** 10.3389/fonc.2020.01414

**Published:** 2020-08-11

**Authors:** Liang Liu, Zhiqin Zheng, Juan Li, Yuan Li, Jianjiao Ni

**Affiliations:** ^1^Department of Radiation Oncology, Fudan University Shanghai Cancer Center, Shanghai, China; ^2^Department of Oncology, Shanghai Medical College, Fudan University, Shanghai, China; ^3^Department of Radiation Oncology, Minhang Branch Hospital, Fudan University Shanghai Cancer Center, Shanghai, China; ^4^Department of Pathology, Fudan University Shanghai Cancer Center, Shanghai, China

**Keywords:** supraclavicular recurrence, postoperative radiotherapy, non-small cell lung cancer, overall survival, clinical target volume

## Abstract

**Background:** The clinical value and delineation of clinical target volume (CTV) of postoperative radiotherapy (PORT) in completely resected (y)pN2 non-small cell lung cancer (NSCLC) remain controversial. Investigations specifically focusing on the cumulative incidence and prognostic significance of initial disease recurrence at the supraclavicular region (SCR) in this disease population are seldom reported.

**Methods:** Consecutive patients with curatively resected (y)pN2 NSCLC who received adjuvant chemotherapy from January 2013 to December 2018 at our cancer center were retrospectively examined. Disease recurrence at the surgical margin, ipsilateral hilum, and/or mediastinum was defined as loco-regional recurrence (LRR). Disease recurrence beyond LRR and SCR, was defined as distant metastasis (DM). Overall survival (OS1 and OS2) were calculated from surgery and disease recurrence to death of any cause, in the entire cohort and in patients with recurrent disease, respectively.

**Results:** Among the 311 patients enrolled, PORT without elective supraclavicular nodal irradiation (ESRT) was performed in 94 patients and neoadjuvant chemotherapy was administered in 31 patients. With a median follow-up of 26 months, 203 patients developed recurrent disease, including 27 SCRs, among which 16 were without DM and 22 involved the ipsilateral supraclavicular region. The 1, 3, and 5-year cumulative incidence of SCR were 6.53, 13.0, and 24.7%, respectively. Chosen DM as a competing event, cN2, ypN2, not receiving lobectomy, and negative expression of CK7 were significantly associated with SCR using the univariate competing risk analysis, while ypN2 was identified as the only independent risk factor of SCR (*p* = 0.012). PORT significantly reduced LRR (*p* = 0.031) and prolonged OS1 (*p* = 0.018), but didn't impact SCR (*p* = 0.254). Pattern of failure analyses indicated that the majority of LRRs developed within the actuarial or virtual CTV of PORT, and 15 of the 22 ipsilateral SCRs could be covered by the virtual CTV of proposed ESRT. In terms of OS2, patients who developed SCR but without DM had intermediate prognosis, compared with those who had DM (*p* = 0.009) and those who had only LRR (*p* = 0.048).

**Conclusions:** SCR is not uncommon and has important prognostic significance in completely resected (y)pN2 NSCLC. The clinical value of PORT and ESRT in such patients need to be further investigated.

## Introduction

Stage III non-small cell lung cancer (NSCLC) is a heterogeneous disease and surgical resection with or without neoadjuvant therapy could be carried out in selected patients (1, 2). After curative resection, disease recurrence poses a considerable threat and it has been demonstrated that platinum-based adjuvant chemotherapy could significantly reduce postoperative recurrence and improve 5-year survival (3, 4). However, although numerous retrospective studies and several population-based investigations (5–9) have suggested a beneficial role of postoperative radiotherapy (PORT) in reducing loco-regional recurrence (LRR), prolonging disease-free survival (DFS) and even improving overall survival (OS) among patients with completely resected (y)pN2 NSCLC (5, 10, 11), the clinical value of PORT is still controversial due to a lack of convincing data from large randomized clinical trials (12, 13).

Moreover, there is no definite agreement on the delineation of clinical target volume (CTV) during PORT for completely resected (y)pN2 NSCLC, and it varies between different institutions and clinical trials (14, 15). The rationales of CTV delineation are mostly based on the patterns of disease recurrence in surgical resected patients who don't receive PORT and patterns of treatment failure in those who receive PORT. In these studies, cumulative incidence, anatomic locations, and risk factors of LRR were extensively examined. However, the definitions of LRR are different, some of which include the initial disease recurrence developed in the supraclavicular region (SCR) (9, 16), while others don't (7, 17). Investigations specifically focused on SCR are seldom reported and elective supraclavicular nodal irradiation (ESRT) is not routinely performed.

In the current study, we investigated the cumulative incidence, risk factor, and prognostic significance of SCR in completely resected (y)pN2 NSCLC. Additionally, our recent study finds crucial prognostic value of routine immunohistochemical (IHC) markers in completely resected NSCLC (18). Hence, besides common clinic-pathological variables, a list of routine IHC markers were examined when investigating the risk factors of SCR.

## Materials and Methods

### Patients

Lung cancer patients who received surgery at Fudan University Shanghai Cancer Center (FUSCC) from January 2013 to December 2018 were retrospectively reviewed. Patients who underwent complete surgical resection (19), with pathologically confirmed N2 disease and received standard adjuvant chemotherapy, were included in the study. Patients received PORT or not, as well as neoadjuvant chemotherapy or not, were both allowed to be included. Exclusion criteria included a second primary tumor, compromised resection, positive surgical margins, neoadjuvant radiotherapy, receiving no adjuvant chemotherapy, death due to surgical complications, and postoperative follow up <3 months.

For each patient, common clinic-pathological parameters were gathered from the electronic medical records, including age, sex, smoking history, the Eastern Corporative Oncology Group (ECOG) performance score, clinical TNM stage, pathological TNM stage, primary tumor size, tumor differentiation, tumor histology, tumor location, lymphovascular invasion, visceral pleural invasion, perineural invasion, and type of surgery. Pathologic TNM stage was in accordance with the eighth edition Lung Cancer Stage Classification (20). Tumor differentiation and tumor histology were determined on the basis of the 2015 World Health Organization Classification of Tumors of the Lung, Pleura, Thymus, and Heart (21). Besides, the expression status of 12 IHC markers (i.e., HER2, TTF1, ERCC1, CK20, CK5/6, CK7, P63, NapsinA, Syn, RRM1, EGFR, and Ki67) were collected. The IHC staining and evaluation were routinely performed in the Immunohistochemistry Diagnostic Laboratory of our cancer center. Our study followed The Declaration of Helsinki. The institutional review board of FUSCC approved the study. Informed consent was waived by the institutional review board because this was a retrospective study.

### Treatment

Pretreatment evaluation generally included clinical assessment, blood test, bronchoscopy, contrast-enhanced chest computed tomography (CT) scan, ultrasonographic examination or CT scan of the abdomen, brain magnetic resonance imaging (MRI) and bone scans. Patients with mediastinal lymph node enlargement (>1 cm) in the short axis on CT scan or pathologically proven to be malignant, were defined as harboring clinical N2 (cN2) disease. Of note, positron emission tomography (PET)/CT, as well as invasive staging of the mediastinum, was strongly recommended for patients with cN2 disease at our cancer center.

Neoadjuvant therapy generally consisted of 3–4 cycles of platinum-based doublet regimen and surgical treatment included lobectomy, sublobectomy, and pneumonectomy, with systematic multilevel mediastinal lymph node dissection or adequate mediastinal sampling (no <3 N2 stations, must include the subcarinal station). PORT was performed according to our institutional protocol (7), using the intensity-modulated radiation therapy technique employing a linear accelerator with 6-MV X-rays. Briefly, the CTV for left lung cancers included the bronchial stump and 2R, 2L, 4R, 4L, 5, 6, 7, 10, and 11L lymph node stations, while the CTV for right lung cancers included the bronchial stump and 2R, 4R, 7, 10, and 11R stations. ESRT was not performed. The total radiation dose prescibed to the planning target volume (PTV) was generally 50.4 Gy, administered daily at 1.8 Gy per fraction, 5 days per week.

### Follow Up

Follow-ups were at the discretion of the treating physicians and were generally scheduled at regular intervals: every 3 months after surgery in the first 2 years, every 6 months for the next 3 years and annually thereafter. During follow-up, blood tests, chest CT scans, and CT scans or ultrasonographic examination of abdominal and cervical regions, were routinely performed, while brain MRI and bone scans were not mandatory. Telephone calls were also implemented when necessary.

Postoperative recurrence was diagnosed considering all the evidence provided by imaging scans and pathologic confirmation. Initial disease recurrence in the supraclavicular region was defined as SCR and first relapse developed at the surgical margin, ipsilateral hilum, and/or mediastinum was considered LRR. Initial disease recurrence beyond LRR and SCR, was categorized as distant metastasis (DM).

### Pattern of Failure Analyses

For patients with LRR, the PTVs were restored for those who received PORT and virtual PTVs were created for those who didn't receive PORT by independent radiation oncologist, according to our institutional protocol mentioned above. Meanwhile, for patients with SCR, individual virtual PTVs were created for ipsilateral ESRT (PTV-sc) by independent radiation oncologist, according to the CT atlas proposed by Lynch et al. (22). Then, we plotted the sites of LRRs and/or SCRs, and overlaid them with restored or created PTVs. Coverage of the LRRs and SCRs by the PTVs were investigated.

### Statistical Analyses

Recurrence free survival (RFS) was calculated from surgery to initial disease recurrence. Overall survival (OS1) was calculated from surgery to death of any cause in the entire cohort and OS2 was calculated from initial disease recurrence to death of any cause in patients with recurrent disease. Differences between clinical parameters were compared using the χ^2^ and Fisher's exact tests. The predictors of SCR were selected using competing risk methodology and Stata version 13.1 software (StataCorp, College Station, TX, USA). The associations between clinic-pathological parameters and OS were identified using the Cox proportional hazard regression model. The hazard ratio (HR) and the 95% confidence interval (CI) were calculated using coefficients from the model. Kaplan–Meier method was used to estimate survival, and differences among groups were investigated by the log-rank test. Statistical analysis was performed using SPSS 21.0 (SPSS, Chicago, IL, USA). All assessment is considered to be significant when two-sided *p*-value is <0.05.

## Results

### Patients Characteristics

A total of 311 patients were finally enrolled and a flowchart for patient selection was presented in Supplementary Figure 1. Detailed baseline disease characteristics of the 311 patients were summarized in Table 1. The majority of patients had a histology of non-squamous NSCLC and received lobectomy. The positive rate of HER2, TTF1, ERCC1, CK20, CK5/6, CK7, P63, NapsinA, Syn, RRM1, and EGFR, was 31.8, 64.0, 39.9, 6.4, 25.4, 80.4, 38.6, 54.3, 12.5, 45.0, and 60.8%, respectively. Additionally, Ki67 ≥ 50% was detected in 43.7% of the patients. Pretreatment PET/CT was performed in 237 (76.2%) patients and invasive staging of the mediastinum was underwent in 35 (11.3%) patients. One hundred and sixty-four patients were found to have cN2 disease, among whom 148 (90.2%) patients received pretreatment PET/CT and 30 (18.3%) patients had invasive staging of the mediastinum. A total of 31 (18.9%) patients received neoadjuvant chemotherapy.

**Table 1:** Disease characteristics.

**Variables**	**Number of patients (%)**
**Age at diagnosis (years)**	
≤ 65	151 (48.6)
>65	160 (51.4)
**Sex**	
Female	127 (40.8)
Male	184 (59.2)
**Smoking history**	
Ever smoker	144 (46.3)
Never smoker	167 (53.7)
**ECOG performance score**	
0	252 (81.0)
1	59 (19.0)
**Clinical N stage**	
cN0–1	147 (47.3)
cN2	164 (52.7)
**Neoadjuvant chemotherapy**	
Yes	31 (10.0)
No	280 (90.0)
**Surgery type**	
Sublobar	18 (5.8)
Lobectomy	276 (88.7)
Pneumonectomy	17 (5.5)
**Pathological T stage**	
pT0–2	262 (84.2)
pT3–4	49 (15.8)
**Lymphovascular invasion**	
Absent	150 (48.2)
Present	161 (51.8)
**Visceral pleural invasion**	
Absent	195 (62.7)
Present	116 (37.3)
**Tumor location**	
Left lower lobe	44 (14.1)
Left upper lobe	90 (29.0)
Right lower lobe	53 (17.0)
Right middle lobe	44 (14.1)
Right upper lobe	80 (25.7)
**Histology**	
Squamous cell carcinoma	61 (19.6)
Non-squamous non-small cell lung cancer	250 (80.4)

*ECOG, Eastern Corporative Oncology Group*.

### Cumulative Incidence and Risk Factors of SCR

**Figure 1:**
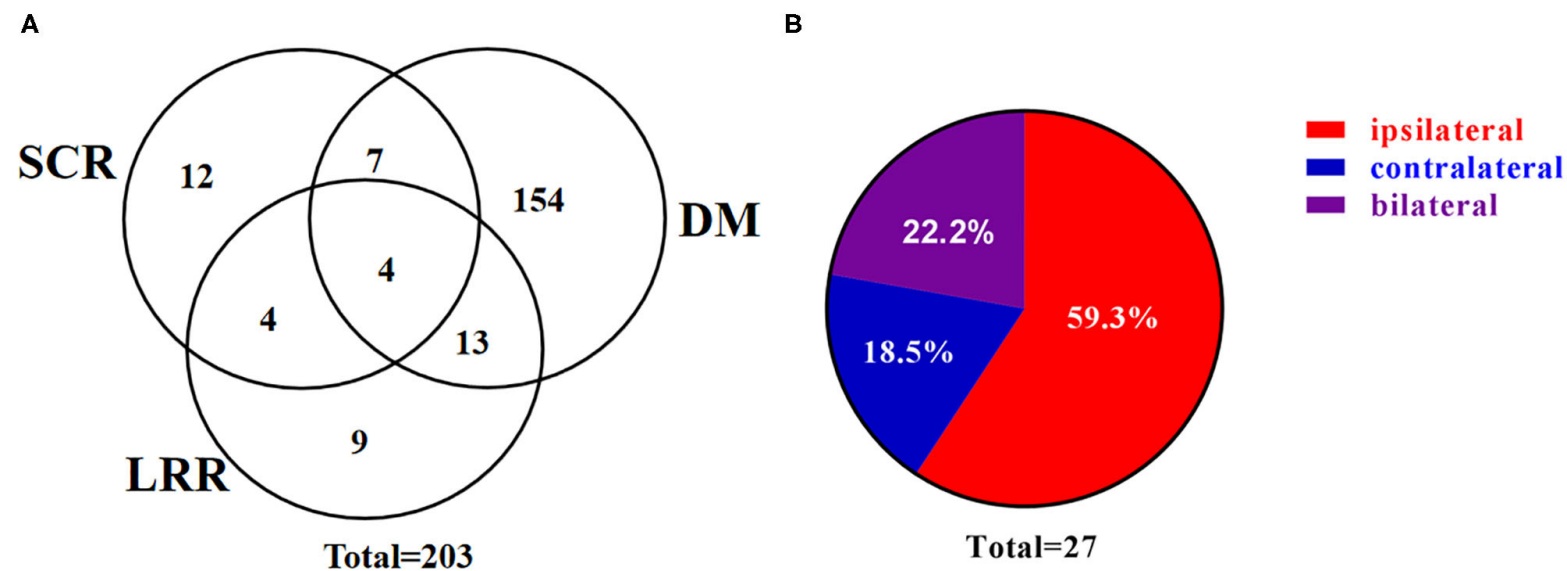
Patterns of supraclavicular recurrence. **(A)** Venn diagram demonstrating the distribution of initial postoperative recurrence. **(B)** Pie chart demonstrating the distribution of SCR. SCR, supraclavicular recurrence; LRR, loco-regional recurrence; DM, distant metastasis.

Post surgery, 94 patients received PORT and with a median follow up of 26 (range, 3–78) months, 203 patients developed recurrent disease, including 27 SCRs. Of note, 17 of the 27 SCRs were pathologically confirmed and the rest 10 were diagnosed by clinical assessments and radiographic findings. The 1, 3, and 5-year RFS were 56.9, 23.9, and 9.0%, in patients without PORT, respectively, and were 71.5, 42.7, and 27.4%, in patients with PORT, respectively. Among the 27 patients with SCR, 16 (59.3%) patients developed SCR without DM (Figure 1A) and 22 (81.5%) patients developed SCR involving the ipsilateral supraclavicular region (Figure 1B). Moreover, among the 12 patients with left-lung cancer who developed SCR, seven were ipsilateral, three bilateral, and two contralateral. Among the 15 patients with right-lung cancer who developed SCR, nine were ipsilateral, three bilateral, and three contralateral.

**Figure 2:**
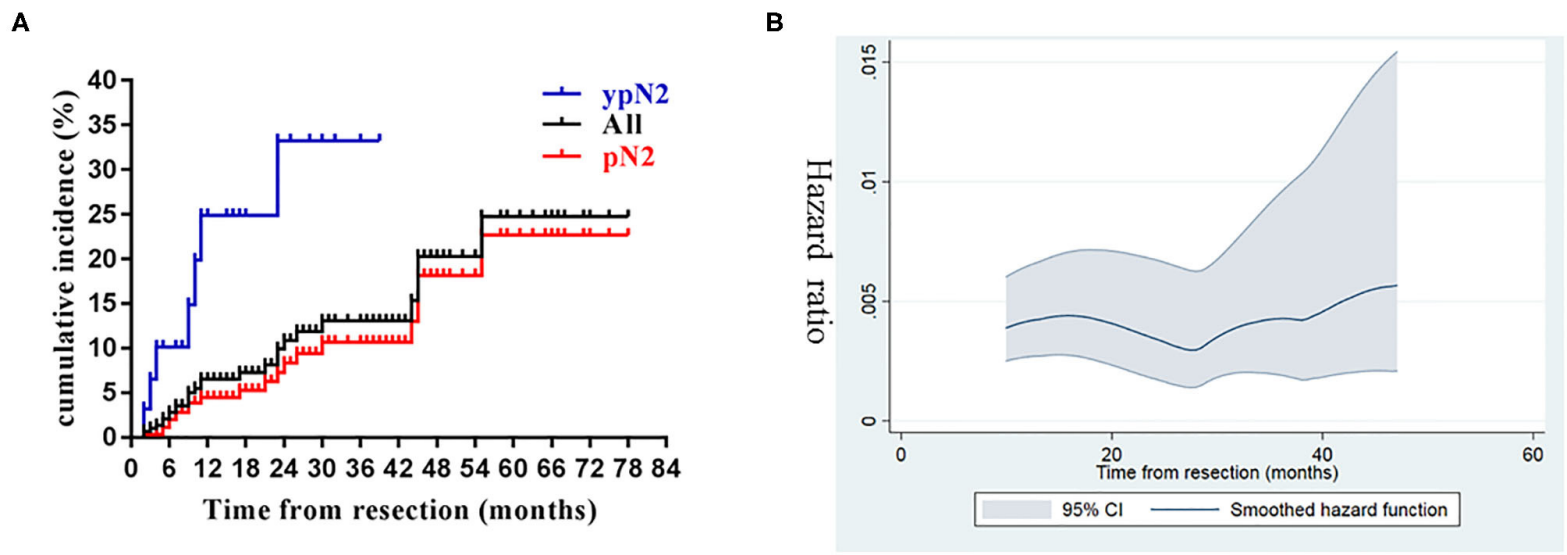
Cumulative incidence and dynamics of supraclavicular recurrence. **(A)** Cumulative incidence of supraclavicular recurrence in the entire cohort and stratified by pathological status (ypN2 vs. pN2). **(B)** The dynamics of hazard ratio of supraclavicular recurrence.

The 1, 3, and 5-year cumulative incidence of SCR were 6.53, 13.0, and 24.7%, respectively (Figure 2A), and the dynamic of hazard ratio of SCR was presented in Figure 2B. Chosen DM as a competing event, cN2 disease, ypN2, lobectomy, and CK7 were identified as significant risk factors of SCR using the univariate competing risk analysis (Table 2). Since there was a significant association between cN2 disease and receiving neoadjuvant chemotherapy (*p* <0.001, χ^2^ test), we excluded cN2 disease and included the other three significant risk factors in the multivariate competing risk analyses. The result showed that only ypN2 were identified as an independent risk factor of SCR (Table 2). The 1, 3, and 5-year cumulative incidence of SCR were 24.90, 33.24, and 33.24% among ypN2 patients, respectively, and were 4.46, 10.67, and 22.69% among pN2 patients, respectively (Figure 2A).

**Table 2:** Competing risk analyses of clinical-pathological variables associated with supraclavicular recurrence.

**Variables**	**Univariate Analyses**		**Multivariate Analyses**	
	**HR (95%CI)**	***p***	**HR (95%CI)**	***p***
Age (>65 vs. ≤ 65)	0.85 (0.40–1.81)	0.671		
Sex (Male vs. Female)	1.23 (0.56–2.69)	0.604		
Smoking (Never vs. Ever)	0.77 (0.35–1.69)	0.577		
ECOG (1 vs. 0)	1.81 (0.62–5.26)	0.279		
cN2 (+ vs. –)	2.58 (1.09–6.11)	**0.031**		
pT stage (T3–4 vs. T0–2)	0.72 (0.22–2.41)	0.599		
pN1 (+ vs. –)	0.83 (0.39–1.80)	0.643		
Multiple levels of pN2 (+ vs. –)	1.13 (0.53–2.42)	0.745		
Histology (SCC vs. Non-SCC)	1.24 (0.50–3.07)	0.646		
Differentiation (P vs. W/M)^&^	1.23 (0.57–2.65)	0.603		
LVI (+ vs. –)	1.54 (0.72–3.31)	0.271		
VPI (+ vs. –)	1.09 (0.49–2.43)	0.836		
PNI (+ vs. –)	0.86 (0.20–3.64)	0.834		
ypN2 vs. pN2	4.61 (1.89–11.22)	**0.001**	3.32 (1.30–6.50)	**0.012**
Tumor Location (Left vs. Right)	0.81 (0.38–1.73)	0.582		
Tumor Lobe (Upper vs. Others)	1.25 (0.78–5.23)	0.374		
TLN (≥16 vs. <16)	0.60 (0.28–1.29)	0.190		
PLN (≥3 vs. <3)	0.95 (0.44–2.02)	0.885		
LNR (≥0.2 vs. <0.2)	1.88 (0.84–4.19)	0.123		
Surgery (Others vs. Lobectomy)	3.68 (1.61–8.42)	**0.002**	1.32 (0.76–2.39)	0.319
PORT (+ vs. –)	0.62 (0.27–1.43)	0.260		
ERCC1 (+ vs. –)	0.90 (0.53–1.52)	0.695		
Her2 (+ vs. –)	1.10 (0.67–1.78)	0.715		
Ki67 (≥50 vs. <50%)	1.81 (0.84–3.88)	0.129		
TTF1 (+ vs. –)	0.64 (0.33–1.24)	0.181		
CK20 (+ vs. –)	1.07 (0.67–1.70)	0.784		
CK7 (+ vs. –)	0.32 (0.15–0.68)	**0.003**	0.46 (0.19–1.13)	0.090
CK5/6 (+ vs. –)	1.24 (0.77–2.01)	0.180		
P63 (+ vs. –)	1.13 (0.66–1.93)	0.651		
NapsinA (+ vs. –)	0.91 (0.50–1.64)	0.754		
Syn (+ vs. –)	1.23 (0.71–2.57)	0.326		
RRM1 (+ vs. –)	0.99 (0.58–1.69)	0.955		
EGFR (+ vs. –)	0.98 (0.54–1.79)	0.946		

HR, hazard ratios; CI, confidence intervals; SCC, squamous cell carcinoma; LVI, Lymphovascular invasion; VPI, Visceral pleural invasion; PI, perineural invasion; ECOG, the Eastern Corporative Oncology Group; TLN, total lymph node examined; PLN, positive lymph node; LNR, positive lymph node ratio; PORT, postoperative radiotherapy;

& *W/M, well/moderate; P, poor. Bold values indicates statistical significant*.

### Pattern of Failure Analyses

**Figure 3:**
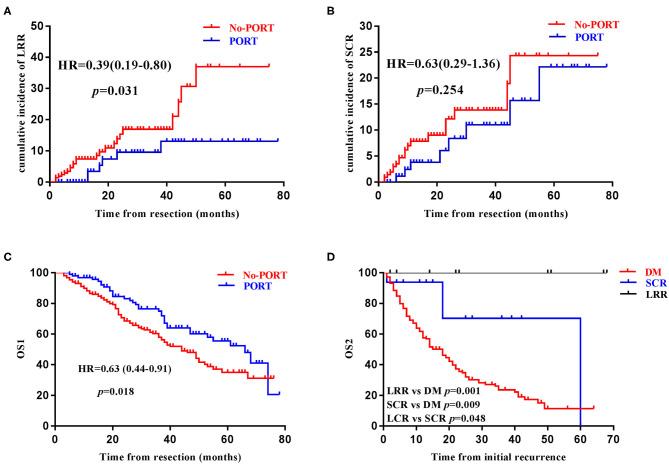
Prognostic significance of postoperative radiotherapy and supraclavicular recurrence. The impact of postoperative radiotherapy (PORT) on loco-regional recurrence (LRR) **(A)**, supraclavicular recurrence (SCR) **(B)**, overall survival (OS1) **(C)**, in the entire cohort. Kaplan–Meier survival curve stratified by the disease recurrence patterns among patients with recurrent disease **(D)**.

In the entire cohort, 6 (6.38%) of the 94 patients who received PORT developed LRR, while 24 (11.1%) of the 217 patients who did not receive PORT developed LRR. PORT significantly reduced the risk of LRR (Figure 3A). Among the six patients who received PORT and subsequently developed LRR, five developed LRR only within the PTV and the rest one developed LRR both within and outside the PTV. Among the 24 patients who did not receive PORT and subsequently developed LRR, 20 developed LRR only within the proposed PTV, three developed LRR both within and outside the proposed PTV, and the rest one developed LRR outside the proposed PTV. That patient had adenocarcinoma in the middle lobe of right lung, with pathologically proven metastatic lymph node in the right hilum and station 7, but developed recurrent disease at mediastinal lymph node stations 5 and 6.

On the other hand, 8 (8.51%) of the 94 patients who received PORT developed SCR, while 19 (8.76%) of the 217 patients who did not receive PORT developed SCR in the entire cohort. PORT without ESRT didn't reduce the incidence of SCR (Figure 3B). Fifteen of the 16 ipsilateral SCRs could be covered by the proposed PTV-sc and the ipsilateral parts of the six bilateral SCRs could all be covered by the proposed PTV-sc.

### Survival Analyses

By the time of data cut-off, 125 patients had died and the median OS1 was 49.0 (95CI 40.5–57.6) months. PORT was found to significant prolong OS1 in the entire cohort (Figure 3C). Age, sex, ECOG score, lymphovascular invasion, total number of positive lymph node, positive lymph node ratio, PORT, and Ki67, were found to be significantly associated with OS1 in univariate Cox analyses, while age, ECOG score, PORT, and Ki67 were identified to be independent indicators of OS1 in multivariate Cox analyses (Table 3). Among the 203 patients with recurrent disease, the median OS2 was 19.0 (95CI 14.7–23.3) months. Age, sex, ECOG score, and DM were revealed to be significantly associated with OS1 in univariate and multivariate Cox analyses (Table 4).

**Table 3:** Cox analyses of clinical-pathological variables associated with overall survival (OS1).

**Variables**	**Univariate Analyses**		**Multivariate Analyses**	
	**HR (95%CI)**	***p***	**HR (95%CI)**	***p***
Age (>65 vs. ≤ 65)	1.64 (1.14–2.15)	**0.008**	1.46 (1.01–2.12)	**0.048**
Sex (Male vs. Female)	1.49 (1.03–2.17)	**0.035**	1.40 (0.94–2.08)	0.097
Smoking (Never vs. Ever)	1.21 (0.85–1.73)	0.285		
ECOG (1 vs. 0)	2.67 (1.71–4.19)	**0.001**	2.22 (1.41–3.52)	**0.001**
cN2 (+ vs. –)	1.11 (0.78–1.57)	0.575		
pT stage (T3–4 vs. T0–2)	1.19 (0.74–1.90)	0.473		
pN1 (+ vs. –)	1.51 (1.01–2.25)	0.042		
Multiple levels of pN2 (+ vs. –)	1.18 (0.83–1.67)	0.366		
Histology (SCC vs. Non-SCC)	1.35 (0.88–2.08)	0.167		
Differentiation (P vs. W/M)^&^	1.05 (0.80–1.24)	0.899		
LVI (+ vs. –)	1.49 (1.04–2.12)	**0.028**	1.21 (0.84–1.75)	0.297
VPI (+ vs. –)	1.29 (0.90–1.85)	0.168		
PNI (+ vs. –)	1.22 (0.70–2.14)	0.477		
ypN2 vs. pN2	0.76 (0.37–1.55)	0.445		
Tumor Location (Left vs. Right)	0.80 (0.56–1.14)	0.217		
Tumor Lobe (Upper vs. Others)	1.17 (0.89–1.25)	0.913		
TLN (≥16 vs. <16)	0.92 (0.63–1.33)	0.644		
PLN (≥3 vs. <3)	1.46 (1.02–2.10)	**0.041**	1.33 (0.80–2.21)	0.274
LNR (≥0.2 vs. <0.2)	1.44 (1.01–2.07)	**0.048**	1.29 (0.78–2.13)	0.324
Surgery (Others vs. Lobectomy)	0.80 (0.43–1.49)	0.483		
PORT (+ vs. –)	0.63 (0.42–0.93)	**0.020**	0.61 (0.40–0.92)	**0.018**
ERCC1 (+ vs. –)	1.03 (0.80–1.32)	0.825		
Her2 (+ vs. –)	0.92 (0.72–1.16)	0.464		
Ki67 (≥50 vs. <50%)	1.65 (1.16–2.34)	**0.006**	1.55 (1.06–2.25)	**0.023**
TTF1 (+ vs. –)	0.81 (0.59–1.10)	0.181		
CK20 (+ vs. –)	0.96 (0.76–1.20)	0.704		
CK7 (+ vs. –)	0.75 (0.49–1.14)	0.178		
CK5/6 (+ vs. –)	1.07 (0.85–1.35)	0.545		
P63 (+ vs. –)	1.02 (0.80–1.32)	0.857		
NapsinA (+ vs. –)	0.79 (0.60–1.05)	0.103		
Syn (+ vs. –)	0.521 (0.23–1.41)	0.324		
RRM1 (+ vs. –)	0.891 (0.62–1.42)	0.897		
EGFR (+ vs. –)	0.82 (0.62–1.08)	0.155		

HR, hazard ratios; CI, confidence intervals; SCC, squamous cell carcinoma; LVI, Lymphovascular invasion; VPI, Visceral pleural invasion; PI, perineural invasion; ECOG, the Eastern Corporative Oncology Group; TLN, total lymph node examined; PLN, positive lymph node; LNR, positive lymph node ratio; PORT, postoperative radiotherapy;

& *W/M, well/moderate; P, poor. Bold values indicates statistical significant*.

**Table 4:** Cox analyses of clinical-pathological variables associated with OS2 in patients with recurrent disease.

**Variables**	**Univariate Analyses**		**Multivariate Analyses**	
	**HR (95%CI)**	***p***	**HR (95%CI)**	***p***
Age (>65 vs. ≤ 65)	1.70 (1.17–2.47)	**0.005**	1.49 (1.02–2.19)	**0.040**
Sex (Male vs. Female)	1.57 (1.07–2.30)	**0.020**	1.74 (1.19–2.56)	**0.005**
Smoking (Never vs. Ever)	1.23 (0.86–1.77)	0.260		
ECOG (1 vs. 0)	1.95 (1.22–3.10)	**0.005**	2.02 (1.25–3.26)	**0.004**
cN2 (+ vs. –)	1.12 (0.78–1.61)	0.534		
pT stage (T3–4 vs. T0–2)	1.15 (0.71–1.86)	0.572		
pN1 (+ vs. –)	1.11 (0.74–1.65)	0.622		
Multiple levels of pN2 (+ vs. –)	0.89 (0.62–1.27)	0.517		
Histology (SCC vs. Non-SCC)	1.56 (0.99–2.42)	0.051		
Differentiation (P vs. W/M)^&^	1.04 (0.71–1.51)	0.851		
LVI (+ vs. –)	1.35 (0.94–1.94)	0.104		
VPI (+ vs. –)	1.07 (0.74–1.55)	0.732		
PNI (+ vs. –)	0.96 (0.55–1.67)	0.875		
ypN2 vs. pN2	0.56 (0.28–1.16)	0.118		
Tumor Location (Left vs. Right)	1.05 (0.73–1.50)	0.810		
Tumor Lobe (Upper vs. Others)	1.03 (0.79–2.17)	0.874		
TLN (≥16 vs. <16)	0.85 (0.65–1.42)	0.849		
PLN (≥3 vs. <3)	1.14 (0.78–1.65)	0.485		
LNR (≥0.2 vs. <0.2)	1.03 (0.72–1.50)	0.361		
Surgery (Others vs. Lobectomy)	1.45 (0.76–2.77)	0.266		
PORT (+ vs. –)	0.85 (0.66–1.23)	0.414		
DM (+ vs. –)	6.49 (2.36–17.85)	** <0.001**	7.43 (2.67–20.68)	** <0.001**
ERCC1 (+ vs. –)	1.04 (0.81–1.33)	0.765		
Her2 (+ vs. –)	0.83 (0.65–1.07)	0.152		
Ki67 (≥50 vs. <50%)	1.19 (0.83–1.70)	0.356		
TTF1 (+ vs. –)	0.73(0.53–1.02)	0.061		
CK20 (+ vs. –)	0.92 (0.72–1.16)	0.472		
CK7 (+ vs. –)	0.87 (0.63–1.20)	0.403		
CK5/6 (+ vs. –)	1.01 (0.80–1.27)	0.955		
P63 (+ vs. –)	0.91 (0.71–1.18)	0.484		
NapsinA (+ vs. –)	0.76 (0.57–1.02)	0.063		
Syn (+ vs. –)	1.37 (0.92–2.35)	0.781		
RRM1 (+ vs. –)	0.97 (0.71–1.42)	0.971		
EGFR (+ vs. –)	0.84 (0.64–1.11)	0.226		

OS, overall survival; HR, hazard ratios; CI, confidence intervals; SCC, squamous cell carcinoma; LVI, Lymphovascular invasion; VPI, Visceral pleural invasion; PI, perineural invasion; ECOG, the Eastern Corporative Oncology Group; TLN, total lymph node examined; PLN, positive lymph node; LNR, positive lymph node ratio; PORT, postoperative radiotherapy; DM, distant metastasis;

& *W/M, well/moderate; P, poor. Bold values indicates statistical significant*.

In order to investigate the prognostic significance of SCR, patients with recurrent disease were further divided into three groups: Group A consisted of patients who had DM (*n* = 178), Group B consisted of patients who did not have DM but have SCR (*n* = 16), and Group C consisted of patients who only had LRR (*n* = 9). In terms of OS2, patients in Group B had an intermediate prognosis, when compared with patients in Group A and Group C (Figure 3D).

## Discussion

To the best of our knowledge, this is the first comprehensive study specifically focusing on SCR in completely resected (y)pN2 NSCLC with a relatively large sample size, in the era of modern radiation technique. SCR was not uncommon and had imperative prognostic significance, indicating that treatment modalities able to reduce the incidence of SCR may be beneficial. Additionally, PORT without ESRT significantly reduced LRR and prolonged OS, but did not decrease SCR in our study, suggesting that the clinical value of ESRT may be reconsidered in selected patients with high risks of SCR.

SCR is not uncommon in completely resected (y)pN2 NSCLC, especially among those with extra risk factors. Although there was limited historical data published that could be directly compared, the incidence of SCR in our study was reliable, since the overall recurrence rate and the percentage of SCR among patients with recurrent disease were in accordance with previous findings. The cumulative incidence of postoperative recurrence in the PORT group and non-PORT group, were generally comparable with recent studies (7, 9, 17, 23). Furthermore, studies from our institution (7) and others (16, 24) had reported a similar percentage of SCR among patients with recurrent disease (7.7–11.6% in the literature, 13.3% in our study). Compared with their counterpart, patients staged cN2 or ypN2 generally had a more advanced and aggressive disease, and thus it was reasonable for them to have a higher risk developing disease recurrence, including SCR (17, 25–27). Compared with those receiving lobectomy, patients receiving pneumonectomy generally had a higher tumor burden and those receiving sublobectomy commonly had unfavorable prognostic factors, such as more comorbidities and poorer preoperative lung functions, that made them unsuitable for lobectomy (23, 28). Therefore, patients who didn't receive lobectomy were also at a higher risk developing postoperative recurrence, which is generally consistent with a recent retrospective study using the SEER database (29). In addition, two recent studies found that positive expression of CK7 were associated with more advanced disease and shorter overall survival (30, 31). In our study, distant metastasis was chosen as a competing event and negative expression of CK7 was identified as a risk factor of SCR, which need to be further verified.

Compared with patients developing only LRR and those developing DM, patients developing SCR but without DM had intermediate OS2, highlighting the vital prognostic significance of SCR in curatively resected (y)pN2 NSCLC. The TNM staging system is one of the most powerful indicators of patient's prognosis in NSCLC, among which patients having supraclavicular lymph node metastasis (N3) generally have intermediate prognosis when compared with those having distant metastasis (M1) and those harboring metastatic tumor lesions limited to the ipsilateral hilar (N1) or mediastinal (N2) lymph nodes (20). Similarly, SCR represented an unfavorable sign of subsequent disease metastasis to distant organs and thus was reasonable to have worse prognosis when compared with those who had only LRR. On the other hand, when compared with those who already had DM, patients who had recurrent disease limited to the thoracic region (i.e., LRR and SCR) could be considered as harboring loco-regional disease and may benefit from aggressive loco-regional treatment, as well as systematic therapies, and thus may still have a chance of long-term survival (32). In fact, among the 16 patients with SCR but without DM, the 3-year survival rate exceeded 70% in our study (Figure 3D). However, due to the advancement of adjuvant chemotherapy and PORT, the number of patients who developed localized recurrent disease (i.e., LRR and SCR) was small (16 patients in group B and 9 patients in group C), although a total of 311 patients were enrolled and followed up for a median of 26 months. Hence, the prognostic significance of SCR needed to be interpreted with caution and future investigations with larger sample size and prospective design are warranted.

The clinical value of PORT in completely resected (y)pN2 NSCLC was demonstrated again in our study, but the delineation of CTV remain controversial. In the current study, PORT significantly reduced LRR and improved OS1, which have been demonstrated in various studies (5, 6, 8–11, 16, 17, 23). However, since ESRT was not routinely performed in our cancer institution (7), PORT failed to reduce SCR, indicating that the majority of SCRs represented the outgrowth of subclinical tumor lesions already in the supraclavicular region and were not originated from the loco-regional recurrent disease through lymphatic metastasis. In fact, 19 (70.4%) of the 27 patients with SCR had no LRR in the current study. These data indicated a potential role of ESRT in selected patients with high risks. Actually, for locally advanced NSCLC receiving chemo-radiotherapy, there is no significant difference of patient's survival between those with or without N3 disease (33, 34), highlighting that the treatment efficacy of chemo-radiotherapy in locally advanced NSCLC was largely dependent on the intrinsic biology of the tumor and the prognosis of patients with or without macroscopic supraclavicular tumor lesions seemed similar. PORT with adjuvant chemotherapy has been repeatedly shown to significantly reduce LRR, indicating the beneficial role of adjuvant chemo-radiotherapy in treating microscopic N1/N2 disease. It is possible that adjuvant chemo-radiotherapy (i.e., adjuvant chemotherapy in combination with ESRT) may also play a role in reducing SCR and subsequently improve patient's survival. Furthermore, nearly 70% of the ipsilateral SCRs could be covered with the virtual CTV of ESRT in our study. However, there are also evidence against the use of ESRT for patients with completely resected NSCLC. Elective irradiation of mediastinal, contralateral hilar and supraclavicular lymph nodes failed to improve patient's survival in unresectable stage III NSCLC without clinical N3 disease (35). And pattern of failure analyses of a prospective trial of PORT without ESRT suggested that the use of limited CTV including only the involved lymph node stations and those with a risk of invasion >10%, was associated with acceptable risk of geographic miss (36). Taken together, PORT without ESRT provided significant clinical benefit for patients with completely resected (y)pN2 NSCLC, and the clinical value of ESRT in highly selected patients, for example those with persistent N2 (ypN2) disease after neoadjuvant chemotherapy, need to be further investigated.

Our study also has some limitations. Firstly, since ESRT is not routinely performed in our cancer center, we could not directly examine the clinical value and prognostic significance of ESRT. Secondly, as this was a retrospectively study, treatment decisions and follow-up strategies were at the discretion of the treating physicians. Different neoadjuvant and adjuvant chemotherapy regimens were used and the protocols of follow-up were not identical. Moreover, since brain MRI and bone scans were not mandatory, asymptomatic brain and/or bone metastasis may be underestimated. Despite these limitations, we believe our study provided valuable information about the cumulative incidence and prognostic significance of SCR in completely resected (y)pN2 NSCLC, which may guide better design of adjuvant treatment modalities and individualized surveillance strategies.

## Data Availability Statement

The raw data supporting the conclusions of this article will be made available by the authors, without undue reservation.

## Ethics Statement

The studies involving human participants were reviewed and approved by the institutional review board of Fudan University Shanghai Cancer Center. Written informed consent for participation was not required for this study in accordance with the national legislation and the institutional requirements.

## Author Contributions

LL, ZZ, and JN: conceptualization. LL and ZZ: methodology, validation, and writing—original draft preparation. LL, ZZ, and JL: formal analysis and investigation. LL, ZZ, and YL: resources and data curation. LL and JN: writing—review and editing. All the authors have approved the final manuscript.

## Conflict of Interest

The authors declare that the research was conducted in the absence of any commercial or financial relationships that could be construed as a potential conflict of interest.

## Supplementary Material

Supplementary Figure 1 Flowchart of patient enrollment. NSCLC, non-small cell lung cancer.
