# Supplementary figures and images for: Supraclavicular Recurrence in Completely Resected (y)pN2 Non-Small Cell Lung Cancer: Implications for Postoperative Radiotherapy
Source: Front Oncol. 2020 Aug 11;10:1414. doi: 10.3389/fonc.2020.01414 (PMC7431951; doi:10.3389/fonc.2020.01414)

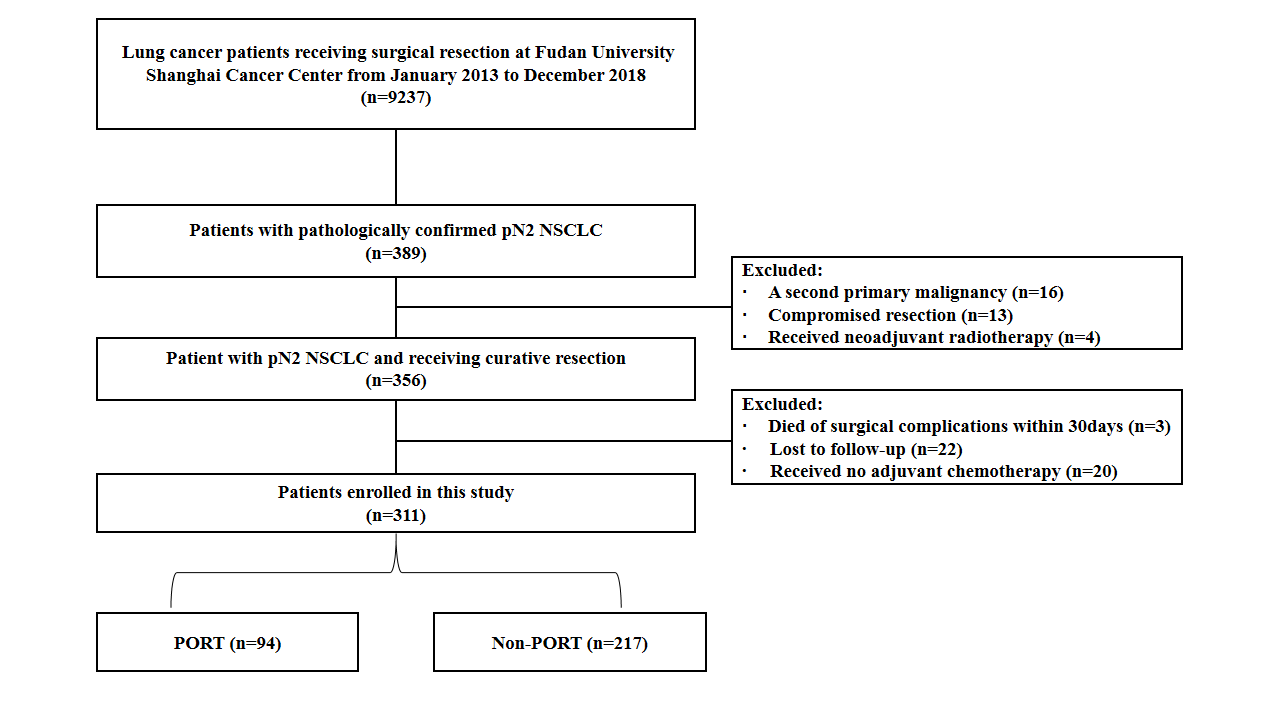

Supplement: Supplementary Figure 1 — Flowchart of patient enrollment. NSCLC, non-small cell lung cancer. [file Image_1.TIF]
